# Evolutionary analysis of human parechovirus type 3 and clinical outcomes of infection during the 2017–18 Australian epidemic

**DOI:** 10.1038/s41598-019-45445-z

**Published:** 2019-06-20

**Authors:** Anthony Chamings, Julian Druce, Leon Caly, Yano Yoga, Philip N. Britton, Kristine K. Macartney, Soren Alexandersen

**Affiliations:** 1 Geelong Centre for Emerging Infectious Diseases, Geelong, Victoria Australia; 2 Deakin University, School of Medicine, Geelong, Victoria Australia; 3 Victorian Infectious Diseases Reference Laboratory (VIDRL), Doherty Institute, Melbourne, Victoria Australia; 4 Marie Bashir Institute, University of Sydney, Sydney, NSW Australia; 5 The Children’s Hospital at Westmead, Sydney, NSW Australia; 6 National Centre for Immunisation Research and Surveillance (NCIRS), Sydney, NSW Australia; 7 Barwon Health, University Hospital Geelong, Geelong, Victoria Australia

**Keywords:** Viral infection, Epidemiology, Whole genome amplification

## Abstract

Human parechovirus type 3 (HPeV3) can cause severe sepsis-like illness in young infants and may be associated with long term neurodevelopmental delay later in childhood. We investigated the molecular epidemiology of HPeV infection in thirty three infants requiring hospitalization before, during and after the peak of the 2017/18 HPeV epidemic wave in Australia. During the peak of the epidemic, all cases were infected with an HPeV3, while before and after the peak, HPeV1 was the predominant type detected. The predominant HPeV3 was the recombinant HPeV3 also detected in the 2013/14 and 2015/16 Australian epidemics. Sepsis-like or meningitis-like symptoms were only reported in cases infected with the recombinant HPeV3. Phylogenetic analysis of the recombinant HPeV3 revealed that the virus continued to evolve, also between the Australian outbreaks, thus indicating continued circulation, despite not being detected and reported in Australia or elsewhere in between epidemic waves. The recombinant HPeV3 continued to show a remarkable stability in its capsid amino acid sequence, further strengthening our previous argument for development of a vaccine or immunotherapeutics to reduce the severity of HPeV3 outbreaks due to this virus.

## Introduction

Human parechovirus (HPeV) is a small non-enveloped RNA virus in the virus family *Picornaviridae*. At least 17 genotypes (HPeV1-17) of human parechovirus have been identified^1^. HPeV infection is often asymptomatic, or associated with mild respiratory or gastrointestinal disease in children. However some types, in particular human parechovirus type 3 (HPeV3), are capable of causing severe disease including central nervous system (CNS) infection and a sepsis-like syndrome in very young children (aged < 3 months)^2–4^. Of children with severe CNS symptoms or sepsis-like disease caused by HPeV3, 25–50% will require intensive care admission^5^. Developmental delay and neurological problems have been observed in up to 50% of children in the 12–18 months after an HPeV3 infection requiring hospitalization^5,6^.

HPeV3 has been identified in several countries in Asia, North America and Europe^5,7–12^. It was first described in 2004 in a sample collected from a young girl in Japan in 1999^7^ and subsequent retrospective studies have found HPeV3 in samples from as early as 1994 in the Netherlands^13^. The epidemiology of HPeV3 appears to be different to other human parechoviruses, with epidemics of infection reported in young children and neonates every 2–3 years^14,15^.

Highly symptomatic cases were recognized in Sydney, Australia, and also detected nationally in 2013/14^16–18^. It was again seen in epidemics in 2015/16^19^ and more recently in 2017/18^4^. The earliest published HPeV3 positive sample in Australia was collected from a sick infant in 2012^20^. This 2012 virus was most similar to an HPeV3 identified in an outbreak in children and adults with myalgia in Yamagata, Japan in 2011^21^. Between 2012 and 2013, a recombination event between a Yamagata-2011 like virus and an unidentified parechovirus occurred forming an Australian recombinant HPeV3. This virus became the predominant HPeV3 identified in the 2013/14 and 2015/16 epidemics in Australia^19,20^.

In Australia, epidemics of HPeV3 have appeared to be larger than those observed in other countries and associated with poorer clinical outcomes^4^. In late 2017 to early 2018, the largest HPeV epidemic to date occurred. A large number of cases were initially seen in Victoria in south eastern Australia, before a similar increase in cases appeared in other states^4^. The objective of this study was to genetically characterize the human parechoviruses involved in the outbreak in south-east Australia in 2017–18, and determine if an association existed between the genotype of the virus and the severity of disease observed in infected infants.

## Results

### Human parechovirus sequencing and typing

Of the 33 clinical cases included in this study, 26 cases were infected with an HPeV3, and 7 cases were infected with an HPeV type 1 (HPeV1) (Table 1: case and sample identifiers). In 24 of the 26 HPeV3 infected infants, the virus detected was closely related to the Australian recombinant HPeV3 identified as the predominant virus in the previous two epidemics in Australia in 2013 and 2015 (see below). Megablast searches of the NCBI GenBank database found that the two remaining HPeV3 cases (case V11 and V12) were most similar to an HPeV3 sequenced in Taiwan in 2011 (GenBank accession: KT626009.1). The HPeV1 sequences were most closely related to a number of different HPeV1’s identified in various parts of the world (Table 1).

**Table 1:** Basic clinical data, sample type, human parechovirus typing results and the most similar HPeV sequence identified by a megaBLAST search of the NCBI Nucleotide database for each case included in this study.

Case	Sample ID	Sample Type	Sample Date	Infant Age (days)	State of Residence	Clinical Syndrome	HPeV Genotype	Complete Polyprotein Sequence	GenBank Accession	Closest Sequence by GenBank Megablast Query	Closest GenBank Accession	GenBank Sequence Year
										**Closest Sequence identified in NCBI Nucleotide Database**		
Case W1	CHW002	CSF	25/11/2017	23	NSW	Sepsis-like	3	Y	MK604037	Australian recombinant HPeV3 FEC10	KY556666.1	2015
Case W2	CHW004	NPA	25/11/2017	62	NSW	Fever + Rash	3	Y	MK604038	Australian recombinant HPeV3 FEC10	KY556666.1	2015
Case W3	CHW005	Faeces	25/11/2017	94	NSW	Fever	3	Y	MK604039	Australian recombinant HPeV3 FEC10	KY556666.1	2015
Case W4	CHW007	Faeces	29/11/2017	83	NSW	Sepsis-like	3	Y	MK604040	Australian recombinant HPeV3 FEC10	KY556666.1	2015
Case W5	CHW009	CSF	30/11/2017	65 (corrected 23)#	NSW	Sepsis-like	3	Y	MK604041	Australian recombinant HPeV3 FEC10	KY556666.1	2015
Case W6	CHW011	CSF	7/12/2017	37	NSW	Sepsis-like	3	Y	MK604042	Australian recombinant HPeV3 FEC10	KY556666.1	2015
Case W7	CHW012	CSF	11/12/2017	55	NSW	Sepsis-like	3	Y	MK604043	Australian recombinant HPeV3 FEC10	KY556666.1	2015
Case W8	CHW014	Faeces	11/12/2017	48	NSW	Fever	3	Y	MK604044	Australian recombinant HPeV3 FEC10	KY556666.1	2015
Case W9	CHW016	Faeces	13/12/2017	129	NSW	Fever	3	Y	MK604045	Australian recombinant HPeV3 FEC10	KY556666.1	2015
Case W10	CHW021	NPA	16/12/2017	115	NSW	Fever	3	Y	MK604046	Australian recombinant HPeV3 FEC10	KY556666.1	2015
Case W11	CHW020	Faeces	16/12/2017	38	NSW	Sepsis-like	3	Y	MK604047	Australian recombinant HPeV3 FEC10	KY556666.1	2015
Case W12	CHW022	Faeces	25/12/2017	42	NSW	Sepsis-like	3	Y	MK604048	Australian recombinant HPeV3 FEC10	KY556666.1	2015
Case W13	CHW025	NPA	6/01/2018	151	NSW	Fever	3	Near complete	MK604049	Australian recombinant HPeV3 FEC10	KY556666.1	2015
Case W14	CHW029	Faeces	22/01/2018	138	NSW	Gastroenteritis	1	N	MK604050	HPeV1 Strain 16-G4, USA	KY645965.1	2016
Case W15	CHW030	Faeces	26/01/2018	80	NSW	Fever	3	Y	MK604051	Australian recombinant HPeV3 FEC10	KY556666.1	2015
Case W16	CHW031	Faeces	27/01/2018	51	NSW	Sepsis-like	3	Y	MK604052	Australian recombinant HPeV3 FEC10	KY556666.1	2015
Case W17	CHW033	Faeces	29/01/2018	113	NSW	Fever	1	N	MK604053	HPeV1 Isolate 7555312, Netherlands	FM178558.1	2003
Case V1	V7121647	Rectal swab	13/03/2017	42	NSW	Sudden death	1	N	MK604054	HPeV1 Isolate 152478, Netherlands	GQ183018.1	2001
Case V2	V7133401	Nasal swab	21/04/2017	291	NSW	No info provided	1*	N	MK604055	HPeV1/Yokohama/38.14 Japan	LC133458.1	2014
Case V3	V7140151	Faeces	16/05/2017	399	VIC	Irritable, gastroenteritis	1	N	MK604056	HPeV1 Strain 131170176, Japan	LC318432.1	2017
Case V4	V7165116	Faeces	13/08/2017	12	VIC	Sepsis-like	3	Y	MK604057	Australian recombinant HPeV3 FEC10	KY556666.1	2015
Case V5	V7169416	CSF	29/08/2017	17	VIC	Sepsis-like, meningitis	3	Y	MK604058	Australian recombinant HPeV3 FEC10	KY556666.1	2015
Case V6	V7176436	Faeces	20/09/2017	48	VIC	Fever, tachycardia	3	Y	MK604059	Australian recombinant HPeV3 FEC10	KY556666.1	2015
Case V7	V7179060	CSF	2/10/2017	10	VIC	Sepsis-like	3	Y	MK604060	Australian recombinant HPeV3 FEC10	KY556666.1	2015
Case V8	V7181489	Nasal Swab	8/10/2017	9	NSW	No info provided	3	Y	MK604061	Australian recombinant HPeV3 FEC10	KY556666.1	2015
Case V9	V7183916	CSF	14/10/2017	23	ACT	Fever, diarrhoea, tachycardia	3	Y	MK604062	Australian recombinant HPeV3 FEC10	KY556666.1	2015
Case V10	V7185174	Blood (leucocytes + plasma)	21/10/2017	53	VIC	Sepsis-like	3	Y	MK604063	Australian recombinant HPeV3 FEC10	KY556666.1	2015
Case V11	V7185593	Faeces	21/10/2017	87	NSW	Fever	3	N	MK604064	TW-03067-2011 HPeV3, Taiwan	KT626009.1	2011
Case V12	V7193255	Faeces	19/11/2017	27	NSW	No info provided	3	N	MK604065	TW-03067-2011 HPeV3, Taiwan	KT626009.1	2011
Case V13	V7197287	Faeces	3/12/2017	158	NSW	No info provided	3	Y	MK604066	Australian recombinant HPeV3 FEC10	KY556666.1	2015
Case V14	V8112082	Faeces	12/02/2018	20	VIC	Fever, diarrhoea	3	Y	MK604067	Australian recombinant HPeV3 FEC10	KY556666.1	2015
Case V15	V8117543	Faeces	5/03/2018	297	VIC	No info provided	1	N	MK604068	HPeV1 TW-71157-2011, Taiwan	KT626008.1	2011
Case V16	V8140914	Bowel contents	30/05/2018	98	VIC	Sudden death	1	N	MK604069	HPeV1 Strain 131170176, Japan	LC318432.1	2017

*VP1 typing was performed at VIDRL instead of GCEID. ^#^Premature infant. Corrected age in parenthesis calculated using 37 weeks gestation as term. Actual age used in statistical calculations.

The Ampliseq panel, as expected, generated the most data when the Australian recombinant HPeV3 was present in a given sample (between 0.2 and 3.8 million reads per sample). The complete coding sequence of the polyprotein was obtained from all but one of the 24 cases (case W13; from which we obtained the full capsid region, complete 2A, 2B, 2C, 3A, 3C and partial 3D polymerase). Partial 5′ UTR and 3′ UTR sequences were also obtained from these samples, except for case W13 where no 3′UTR sequence data was generated. Regions of low coverage (<5 reads) were seen in samples from 9 cases around nucleotides 3600–3800 and/or 4200–4400 (in relation to sequence KY55666.1) and Sanger sequencing was performed to confirm the sequence in these regions.

The complete VP0, VP3 and partial VP1 sequence was obtained from the two Taiwan 2011-like HPeV3 viruses using the Ampliseq method. Twelve thousand and twenty-eight thousand reads were generated for these samples. These reads were concentrated over the structural proteins whereas the non-structural protein genes had very low and sporadic coverage. The nucleotide sequence in these samples was supplemented with Sanger sequencing of the amplicons from the VP1 typing PCR^22^ and a published 3D polymerase PCR^23^.

The Ampliseq panel typically generated the lowest number of reads in samples containing an HPeV1, with four of the samples generating no usable sequence data by this method. These samples were typed using the VP1 typing PCR. In case V2, the VP1 PCR failed to produce an amplicon, and the typing was therefore based on the original VP1 PCR sequencing done on this sample at the Victorian Infectious Disease Reference Laboratory (VIDRL). Sequence fragments of each HPeV1 did not cover exactly the same region of the VP1 coding sequence, so two alignments were created. The first alignment was 421nt long and included sequences from cases V1, V2, V3, V15 and V16. The nucleotide percentage similarity matrix is shown in Supplementary Table 2. Percentage similarity of the sequences ranged from 77 to 99%. The second alignment was 474nt and included sequences from cases W14, W17, V1, V3, V15 and V16. The nucleotide percentage similarity matrix is shown in Supplementary Table 3. Percentage similarity ranged from 77.6 to 98.5%. Only 2 cases were related (V3 and V16 within about 1% differences), while all others were 10% or more different from each other and thus most likely represent separate introductions or multiple circulating lineages separated by several years of evolution.

### 2017/18 HPeV Epidemic in South Eastern Australia

**Figure 1:**
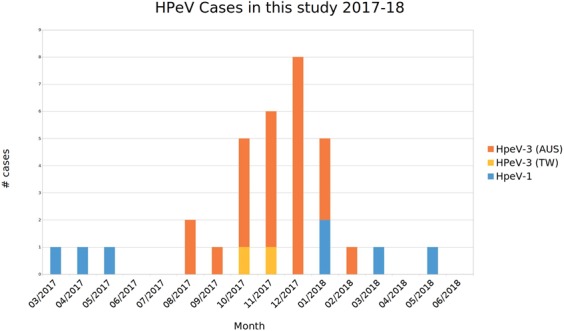
The human parechovirus type present in the samples from sick infants plotted against the month each sample was collected. HPeV type 3 predominated during the height of the 2017–18 parechovirus outbreak in infants in south eastern Australia. HPeV type 1 was detected in samples outside of the epidemic period.

Human parechovirus type 3 was the predominant type detected between August 2017 and January/February 2018, which coincided with a peak number of cases of HPeV in infants in south eastern Australia (Fig. 1). HPeV1 was detected between March and May 2017 and again from late January to May 2018, periods prior to, and following the HPeV3 epidemic.

### Clinical characteristics of HPeV cases in the 2017/18 epidemic

Of the 33 cases included in this investigation, 27 (81%) were male and 6 (19%) were female. The average and median age of infected infants at the time of sampling for HPeV was 81 and 55 days old respectively. The average and median ages of infants infected with HPeV1 were 197 and 138 days respectively, while the average and median ages for infants infected with HPeV3 were only 59 and 50 days respectively. The average and median ages of the HPeV3 infected infants were significantly lower than the HPeV1 cases (p = 0.017, Mann-Whitney U Test).

In 26 of the 33 cases, information on the clinical signs experienced by the infants was available. Meningitis or sepsis-like syndrome were only reported in the children infected with the Australian recombinant HPeV3. However, the association between the type of parechovirus and clinical disease severity (sepsis-like syndrome or not) was not significant (HPeV3: 12/23 cases, HPeV1: 0/3 cases, p = 0.14, Fisher’s Exact Test). HPeV1 was detected in samples from two cases of sudden infant death (Case V1 and V16), however, the ultimate cause of death was not specified and therefore the detection of the HPeV may have been incidental in these cases.

In cases W1 to W17, information on the average duration of stay in hospital was available. Fifteen of these cases were attributed to the Australian recombinant HPeV3 while two were due to infection with HPeV1. The average and median duration of hospitalization for the HPeV3 infected infants was 3.87 and 4 days respectively (range 1–10 days). The average and median duration of the HPeV1 infected infants was 4.5 days (range 4–5 days). However, 8 out of 15 infant HPeV3 cases were listed as having sepsis-like symptoms while the 2 HPeV1 cases (W14 and W17) had less severe gastroenteritis or fever respectively.

### Phylogenetic analysis

Recombination, selection and phylogenetic analysis was performed on the 23 complete polyprotein sequences and partial 5′ and 3′ UTRs from the Australian recombinant type 3 viruses. Other HPeV3 sequences available in GenBank which belonged to this recombinant lineage at the time of writing were also included (Supplementary Table 4). These were 17 HPeV3 sequences from previous Australian HPeV epidemics in 2013/14 and 2015/16^20^.

No evidence of further recombination was detected in the HPeV3 sequences from the Australian epidemics. Selection analysis (Supplementary Table 5) found that the codons in the polyprotein of the Australian recombinant HPeV3 viruses were under negative selection pressure to not change their amino acid sequence, particularly in the structural genes. The REL analysis identified 8 sites in the non-structural genes which were potentially undergoing positive selection, however SLAC and FEL at significance level of p < 0.05, did not identify any sites under positive selection pressure.

**Figure 2:**
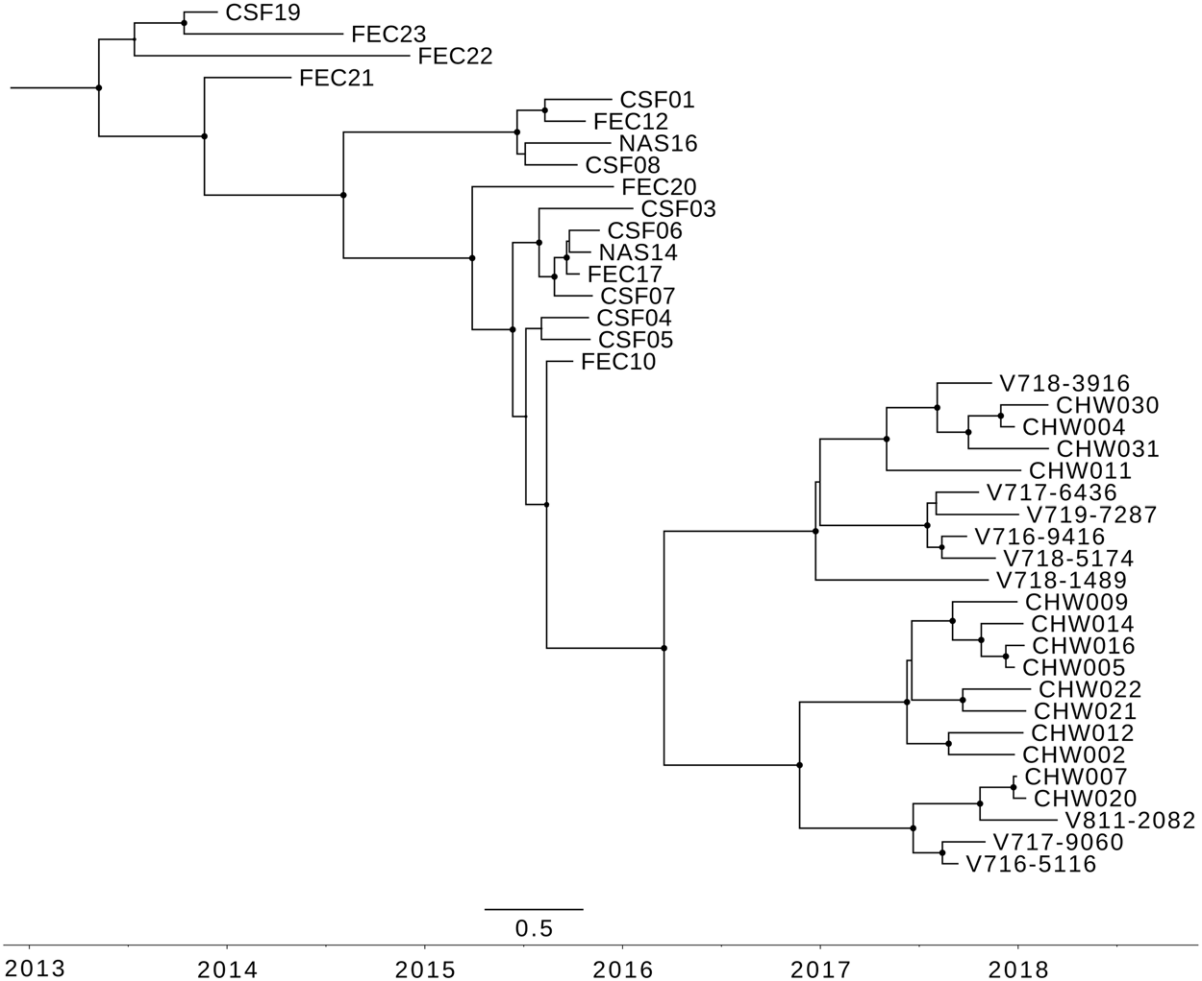
The maximum clade credibility tree of the complete nucleotide sequence (6534 nt) of the polyprotein gene of 23 of the HPeV type 3 viruses detected in the Australian 2017 epidemic and similar viruses identified in the previous Australian epidemics of 2013 and 2015. Sequences were aligned by MUSCLE and phylogenetic analysis was performed in Beast 2.5.2 using the HKY model with a gamma distribution, a Markov Chain Monte Carlo chain length of 50 million and a relaxed evolutionary clock. Node circles are scaled proportional to the posterior probability.

The Bayesian phylodynamic tree for the polyprotein nucleotide sequence is shown in Fig. 2 and the corresponding supporting maximum likelihood phylogenetic tree is shown in Supplementary Fig. 1. The 2017/18 epidemic Australian recombinant HPeV3 viruses were likely descendants of viruses circulating in the 2015 epidemic which in turn were descendants of viruses detected during the 2013/14 epidemic in Australia. The rate of molecular evolution (2.46 × 10^−3^ substitutions per site per year, 95%, CI: 2.00 × 10^−3^ − 2.94 × 10^−3^) was very stable during and between epidemics (see Supplementary Fig. 4) and suggested that there had been ongoing transmission and evolution of this HPeV3 lineage between, as well as during, epidemics.

**Figure 3:**
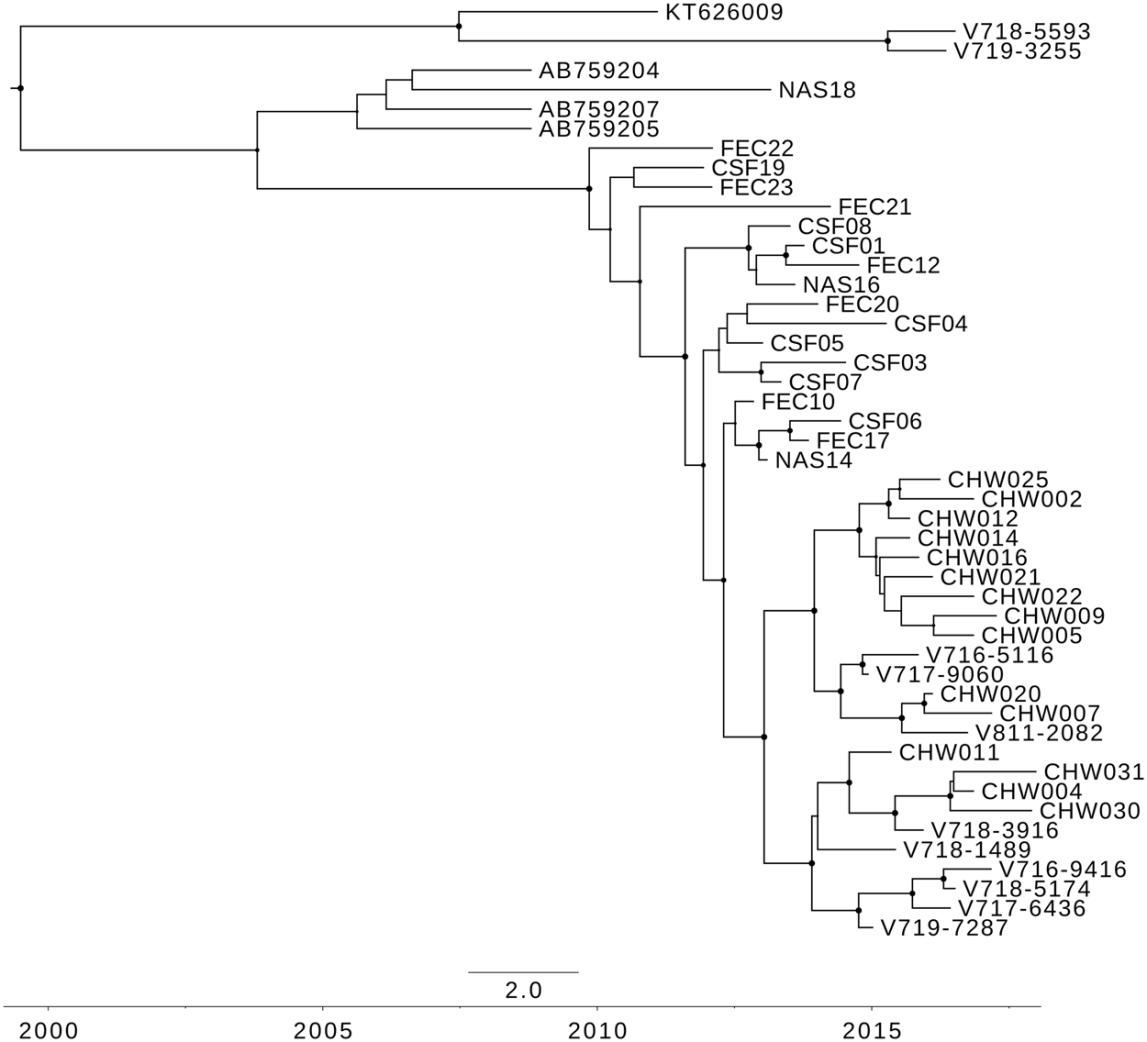
The time scaled Maximum Clade Credibility phylodynamic tree of the complete capsid nucleotide sequence (2313nt) of the HPeV type 3 viruses identified in Australia 2012–2018 and the related representative viruses identified overseas. Sequences were aligned by MUSCLE and phylogenetic analysis was performed in Beast 2.5.2 using the HKY model and a Markov Chain Monte Carlo chain length of 50 million. Node circles are scaled proportional to the posterior probability. The sample name is shown. The structural protein genes of all Australian HPeV type 3’s except V7185593 and V7193255 belonged to a Yamagata-2011 HPeV type 3 lineage.

The capsid gene of the Australian recombinant HPeV3 viruses was previously shown to have likely come from a lineage of viruses related to an HPeV3 detected in Japan in 2011 (Yamagata 2011 lineage)^19^. The maximum clade credibility tree of the capsid nucleotide sequence of all HPeV3 from the Australian epidemics along with their closest counterparts identified overseas (Yamagata 2011 HPeV3 (GenBank accessions: AB759204.1, AB759205.1 and AB759207.1) and Taiwan 2011 (KT626009.1)) is shown in Fig. 3. The maximum likelihood tree of the nucleotide and amino acid sequence of the capsid gene are shown in Supplementary Figs 2 and 3. The nucleotide phylogenetic analysis by both methods indicated that the 2017 recombinant HPeV3 were likely descendants of the HPeV3 viruses circulating during the 2015 epidemic. The two Taiwan 2011-like viruses formed their own branch and had evolved from an ancestor common with KT626009 from Taiwan. The average evolutionary rate in the capsid (9.39 × 10^−4^ nucleotide substitutions per site per year, 95% CI: 6.18 × 10^−4^ − 1.27 × 10^−3^) was lower when compared to the whole polyprotein gene.

This low rate was reflected in the amino acid phylogenetic analysis and in the dN/dS rate (Supplementary Fig. 3 and Table 5). The amino acid sequence of the recombinant HPeV3 capsid proteins varied very little between the 2013, 2015 and 2017 epidemics in Australia. Most virus sequences had identical amino acid sequences, with sequences possessing 1–3 amino acid changes at most.

Within the non-structural proteins of the Australian recombinant HPeV3 viruses, a hotspot of variability was observed in the 2C non-structural gene with codons 1359 and 1366 of the polyprotein changing from polar to basic amino acids in some sequences (codon 1359: asparagine or lysine; codon 1366: glutamine or arginine) (Supplementary Table 6). As these changes could potentially affect the structure/function of the 2C protein, the association between one or two basic amino acids and the clinical observation of sepsis-like syndrome was investigated. Clinical information was available for twenty two of the twenty four cases infected with the Australian recombinant HPeV3. Sepsis-like disease was reported in 5 of the 12 cases where the viruses had 1 basic amino acid at sites 1359 and 1366. Sepsis-like syndrome was observed in 7 cases of the 10 infected with viruses with two basic amino acids. While sepsis-like syndrome was seen in more cases with two basic amino acids, the association was not statistically significant (p = 0.15, Fisher’s exact test).

### Transmission network analysis

The minimum spanning network of the polyprotein nucleotide sequences of the Australian recombinant HPeV3 identified in this study is shown in Supplementary Fig. 5. There was clustering of the recombinant HPeV3 sequences by the year of sampling. There were two distinct networks of sequences observed in the 2015 epidemic. The network indicated that the sequences from the 2017 epidemic were descendants from viruses belonging to just one of these 2015 networks; notably, a group of viruses having two basic amino acids in the 2C gene (see above). Within the 2017 epidemic, there were three subnetworks of cases, and these likely each represented just a small fraction of larger transmission networks. One network was a Sydney-centric outbreak occurring over November and December 2017 (including cases W1, W3, W5, W7, W8, W9, W10, W12). Interestingly, this network included sequences with either one or two basic amino acids in the 2C gene. The other two networks were made up of cases from wider geographic regions and over longer time periods (4 to 7 months) and one of these networks (cases V4, V7, V14 and W4, W11) had viruses with two basic amino acids in 2C and the other network (cases V5, V6, V8, V9, V10 and W2, W6, W15, W16) only a single basic amino acid. Case V8 (Sample V7181489) sat at the end of a long branch in one of the networks, suggesting it was quite different from the next most similar sequence. This one case came from a town over 800 km from Sydney NSW, and was the most geographically isolated sample (See Supplementary Fig. 6). Interestingly, the 2017 virus most closely related to the 2015 ancestors, case V6 (sample V7176436), only had a single basic amino acid at the variable site in 2 C although it appeared to have evolved from the 2015 cluster viruses with 2 basic amino acids. All but one of the Victorian sequences were collected from patients between August and October 2017. The majority of cases from NSW were sampled between November 2017 and January 2018, and this represented the peak of the epidemic in that state to when case numbers started to decrease (Supplementary Fig. 7). The single ACT sequence (V9) was from a sample taken in October 2017.

## Discussion

Human parechovirus type 3 was the most common type of parechovirus identified from cases during the 2017/18 epidemic in south eastern Australia. Viruses belonging to the Australian recombinant HPeV3 lineage were identified in all but two of the HPeV3 cases sampled, making the 2017/18 epidemic the third consecutive Australian epidemic^20^ where this lineage was the predominant virus sequenced from young infants with severe clinical disease.

The phylogenetic analysis indicated that the Australian recombinant lineage of HPeV3 viruses was evolving between epidemics, however the structural protein amino acid sequence was strongly conserved across the epidemics. This could indicate that the advantages conferred by the current capsid structure in infection of host cells outweighed the need to avoid immunological responses and/or the Australian HPeV-3 was not experiencing sufficient selection pressure to change its structural proteins between and during epidemics, perhaps by circulating mainly in young hosts.

Investigators in other countries have observed that the neutralizing antibody titre in sick infants and their mothers were generally lower than healthy controls^24^, and that maternal antibody titres were lower in mothers in their 30’s and 40’s than younger mothers^25^. In 2017, the median age of mothers in Australia was 30.7 years (Australian Bureau of Statistics, 2017 http://www.abs.gov.au/AUSSTATS/abs@.nsf/DetailsPage/3301.02010?OpenDocument). An age associated decrease in neutralizing antibody titres has been observed in the Australian population in people aged over 30 years^12^, and therefore the majority of babies born in Australia may continue to have little protection against HPeV3 during the first 3 months of life when infection, for reasons not yet known, is most problematic.

The majority of the HPeV3 cases were in infants under 3 months of age, while HPeV1 cases were seen in infants older than 3 months. This is consistent with observations by investigators in other countries, where HPeV3 illness is seen most frequently in very young children^3,15,24^. The most severe clinical cases in this study, where meningitis- or sepsis-like syndrome were noted by the treating clinician, were all infected with the Australian recombinant HPeV3 virus. The most noticeable changes to the amino acid sequence of the recombinant HPeV3 viruses occurred at codons 1359 and 1366 of the polyprotein (2C) where some of the viruses exchanged polar amino acids for charged basic amino acids. 70% of cases with a virus with two basic amino acids at these sites developed sepsis-like syndrome, while only 42% of infections with HPeV3 with a single basic amino acid developed sepsis-like disease. While the association was not statistically significant, possibly given the relatively low number of cases in this study, we speculate that these changes may have an effect on the resulting 2C protein structure or function. This may result in a more efficient viral replication complex in infected cells^26^. However, it appears that the evolution of this virus over time has not selected for virus genomes with 2 basic amino acids in the 2C protein, as the 2015 wave already contained viruses with one or two basic amino acids and while the ancestor of the 2017 wave appears to have come from the 2015 cluster of viruses with two basic amino acids at this site, further evolution and selection have produced viruses with two or only one basic amino acids; perhaps suggesting that while two basic amino acids in 2C may be linked to virulence, one basic amino acid in this region of 2C may be of importance for other aspects of virus spread, such as e.g. level of excretion. Further investigation in a larger number of infants combined with *in vitro* studies may better elucidate the role of these amino acid changes.

While the Australian recombinant HPeV3 was the predominant HPeV in the 2017 epidemic, other HPeV’s were detected sporadically. A second HPeV3 most similar to an HPeV3 sequenced in Taiwan in 2011^27^ was identified in two cases during the epidemic. These cases were both from the Hunter Valley region of NSW and are representative of a localized, likely travel-associated, outbreak with this second HPeV3 (case V11 and V12 were from localities ~7 km apart). HPeV1 viruses were also identified, but these were found predominantly in samples from outside the main epidemic period. These may have represented the HPeV types that continuously circulate in the Australian community or were travel related as they were not identical and could have been introduced from different parts of the world. The HPeV1’s did cause clinical disease in the infants, but it appeared to be milder, and sepsis-like syndrome was not seen. Overseas, non-HPeV type 3 parechoviruses are observed to be continuously circulating in children in the community, while HPeV3 has a peak in cases separated by 2–3 years and has been associated with more severe clinical signs^1–3,14,21^.

In the two cases of infant sudden death included in this study, an HPeV1 was detected in gastrointestinal samples, and it was not determined if the HPeV1 played a role in these deaths. However death in children under the age of 2 years associated with HPeV1, HPeV3 and HPeV6 infection have been described in other countries^3,28^. The two cases in our series occurred before and after the main peak in HPeV case numbers during the 2017/18 outbreak. Routine post-mortem testing for HPeV appears warranted when investigating cases of sudden unexplained death in infancy, irrespective of whether an HPeV epidemic is currently occurring or not.

The 2017/18 HPeV epidemic was the largest epidemic by case numbers seen to date in Australia^4^. This study has found that there has been no major genetic changes (ie recombination) in the predominant HPeV3 circulating in Australia, nor has there been a change in the capsid protein structure or antigenicity of the virus circulating that may explain the increased incidence of the disease in 2017. The increase in the number of cases in 2017 compared to earlier epidemics may have been attributable to epidemiological factors such as the virus entering subpopulations with a higher proportion of individuals at risk (young children) such as child care centres, maternal health centres or even maternity wards. The higher number of cases could also be partly due to increased testing for HPeV as awareness about the virus increases in the medical community. More research on the burden of HPeV disease in the community and its epidemiology is certainly warranted, and could be helpful in determining drivers of future epidemics.

The stability of the virus structural proteins again raises the potential that HPeV3 disease may one day be able to be controlled through vaccination of very young babies or pregnant women, and more research into vaccine development and other immunotherapies^29^ could have positive benefits to children in many parts of the world.

## Materials and Methods

### Clinical cases and sample collection

Human parechovirus positive samples from the 2017/18 outbreak in south eastern Australia (NSW and Victoria) were identified by reviewing laboratory and clinical records from the Children’s Hospital at Westmead (CHW), Sydney, New South Wales, and from the laboratory submission database of the Victorian Infectious Disease Reference Laboratory (VIDRL), Melbourne Victoria. Samples were selected for this study on the basis that 1) they had been identified as human parechovirus positive by PCR in a clinical laboratory; and 2) sufficient clinical sample remained for further nucleic acid extraction and sequencing. If multiple HPeV positive samples were available from a clinical case, the sample which gave the lowest C_T_ value (had the highest virus content) in the diagnostic PCR was selected for inclusion in this study. Samples from a total of thirty three cases were ultimately selected for inclusion in this study. The studies performed here were performed in accordance with all relevant guidelines and regulations. Data on samples collected from VIDRL were provided with ethical exemption from the Barwon Health Research Ethics Committee (Ref No. 16/191), data on the clinical presentation of the infants from the Children’s Hospital Westmead was collected with approval from the Sydney Children’s Hospitals Network Human Research Ethics Committee project No. LNR/14/SCHN/528 and Deakin University’s Human Research Ethics Committee project No. 2018–243. Informed consent was obtained using an opt-out process as approved by the Sydney Children’s Hospitals Network Human Research Ethics Committee.

Samples were collected between March 2017 and May 2018 coinciding with the 2017/18 HPeV epidemic in Australia. Samples came from infants which were treated for a range of clinical presentations including irritability, fever, rash, gastroenteritis, meningitis and sepsis-like syndrome. These samples likely represented the most severe cases during the epidemic, where parents sought medical attention for their children. Samples from two cases of sudden infant death (cause of death was not specified) during this period were also included. Sample types included faeces, nasopharyngeal aspirates, blood and cerebrospinal fluid (CSF). Samples were screened for the presence of parechovirus using an established PCR protocol^19^. Infants ranged in age between 7 days to 399 days (1 year and 1 month) and lived in either Victoria, Australian Capital Territory (ACT) or New South Wales, Australia. Basic clinical information on each infant is presented in Table 1. More detailed clinical information was available for cases W1-W17 only. Clinical samples were stored at −80 °C between submission to the diagnostic laboratories and processing for this study.

### Nucleic acid and cDNA synthesis

Nucleic acid from samples submitted to VIDRL was extracted using the Qiagen Qiamp 96 kit and a Qiacube HD extraction robot as per the manufacturer’s instructions (Qiagen, Hilden, Germany). RNA was reverse transcribed using Bioline’s Sensifast synthesis kit (Bioline, UK) as per kit instructions. Nucleic acid from samples collected at CHW was extracted using the Qiagen Viral RNA mini kit (Qiagen, Hilden, Germany) and RNA was reverse transcribed using Life Technologies’ Superscript IV VILO master mix kit (Thermofisher Scientific, Victoria, Australia) as per manufacturers’ instructions. cDNA was stored at −20 °C until processing.

### Next generation sequencing

cDNA from samples was amplified using the Ion Ampliseq™ Library Kit 2.0 (Thermofisher Scientific, Victoria, Australia) and a custom Ampliseq™ primer panel designed based on the sequences of HPeV3 from the 2013 and 2015 HPeV outbreaks as previously described^20^. The protocol was performed as per the manufacturer’s instructions except that 35 PCR cycles were used instead of 30 cycles, and the amplicons from the two primer pools were not combined until after quantification. Library quantification was performed using the Ion Library TaqMan™ Quantification Kit (Thermofisher Scientific).

Libraries were pooled and loaded onto Ion 530 chips and run on an Ion S5 XL genetic sequencer (Thermofisher Scientific) at the Geelong Centre for Emerging Infectious Diseases (GCEID). Eight to thirteen samples were run per chip with an average of 1,057,783 reads generated per sample (Min: 11,962, Max: 3,795,467). Samples with low numbers of reads were repeated.

When regions with low read coverage (<5 reads) were encountered, specific primers were designed and the region sequenced by Sanger sequencing using the BigDye® Terminator v3.1 Cycle Sequencing Kit as per manufacturer’s instructions and a Hitachi 3500XL Genetic sequencer (Thermofisher Scientific). Primers used for Sanger sequencing are provided in Supplementary Table 1.

The consensus sequence from the Ampliseq reads were visualized in Integrative Genomic Viewer^30^ (IGV) (Broad Institute, University of California), and the consensus sequence generated at a Q-score of 80. The consensus sequence and any Sanger sequencing alignments were performed in Geneious 11.1.15 (Biomatters, Auckland, New Zealand) using MUSCLE^31^.

The genotype of the HPeV of each sample was identified by querying the consensus sequence generated from the Ampliseq panel against the Nucleotide database of NCBI using the Basic Local Alignment Search Tool (BLAST) (https://www.ncbi.nlm.nih.gov/BLAST/). When insufficient consensus sequence was available to identify the genotype of the HPeV, a human parechovirus VP1 typing PCR^22^ was performed and the amplicon sequenced to identify the genotype.

### Phylogenetic analysis

HPeV3 sequences were aligned using MUSCLE^31^ along with previous Australian HPeV3 sequences from the 2013 and 2015 epidemics^19^ (see Supplementary Table 4 for GenBank accession details). Screening for recombination was performed using GARD on the Datamonkey^32^ webserver. Codon selection was analysed using the single likelihood ancestor counting (SLAC), fixed effects likelihood (FEL) and random effects likelihood (REL) packages on the Datamonkey webserver. A cut-off p-value of 0.05 was used for SLAC, a p-value of 0.05 and 0.01 for FEL and the cut-off Bayes factor in REL was set to 100 as described previously^20,33^.

The Bayesian Markov Chain Monte Carlo (MCMC) method in Beast 2.5.2^34^ was used to estimate the nucleotiode substitution rate per site per year and the time to the most recent ancestor. The age of each virus was defined as the date of collection. Given the small sample size, the Hasegawa-Kishino-Yano (HKY) model^35^ with a gamma-distributed rate amongst sites was selected. A constant coalescent population and a relaxed clock were used for analysis. Two independent runs using a chain length of 50 million, and sampling every 1000 simulation were conducted for each of the polyprotein, capsid and non-structural gene analyses. The convergence and effective sample size (ESS) of each estimate was checked using Tracer 1.7.1^36^. All parameters for each run showed an ESS >1000. A maximum clade credibility tree was created using TreeAnnotator 2.5.2^34^ to summarize all 50,000 trees after a 10% burn in. Trees were visualized and annotated in FigTree 1.4.4^37^.

Maximum likelihood phylogenetic analysis was performed in MEGA 10.0.4^38^ using the Tamura 3 parameter model^39^ with a gamma-distribution (T92 + G), which was selected as the best model by MEGA for the nucleotide alignments of the polyprotein, capsid and non-structural proteins. 1000 bootstrap replicates were used to test the robustness of the phylogeny. Amino acid maximum likelihood phylogenies were performed using the Jones-Taylor-Thornton substitution model^40^ and 500 bootstrap replicates. All maximum likelihood trees were rooted on the oldest sequence to compare topology with the Bayesian methods.

### Transmission network construction

To understand possible transmission dynamics of HPeV3 in Australia, the minimum spanning network for the nucleotide sequence of the polyprotein HPeV3 sequences from the 2013, 2015 and 2017 Australian epidemics was created in Network v5 (Fluxus-Engineering, Clare, England) with an epsilon value of 10 as described previously^20,33^.

## Supplementary information

Evolutionary analysis of human parechovirus type 3 and clinical outcomes of infection during the 2017‐18 Australian epidemic

## Data Availability

All sequences generated have been deposited in GenBank under accession numbers MK604037-MK604069. Other datasets generated or analysed during the current study are available from the corresponding author on reasonable request.
