# Supplementary material for: Evolutionary analysis of human parechovirus type 3 and clinical outcomes of infection during the 2017–18 Australian epidemic
Source: Sci Rep. 2019 Jun 20;9:8906. doi: 10.1038/s41598-019-45445-z (PMC6586808; doi:10.1038/s41598-019-45445-z)
Supplement: Supplementary file 1 — Evolutionary analysis of human parechovirus type 3 and clinical outcomes of infection during the 2017‐18 Australian epidemic [file 41598_2019_45445_MOESM1_ESM.pdf]

Supplementary Materials

Evolutionary analysis of human parechovirus type 3 and clinical outcomes of infection during the 2017-18 Australian epidemic

Anthony Chamings[1,2], Julian Druce[3], Leon Caly[3], Yano Yoga[3] , Philip N. Britton[4,5], Kristine K. Macartney[4,5,6] and Soren Alexandersen[1,2,7 a]

[1]Geelong Centre for Emerging Infectious Diseases, Geelong, Victoria, Australia;

[2]Deakin University, School of Medicine, Geelong, Victoria, Australia;

[3]Victorian Infectious Diseases Reference Laboratory (VIDRL), Doherty Institute, Melbourne, Victoria, Australia;

[4]Marie Bashir Institute, University of Sydney, Sydney, NSW;

[5]The Children's Hospital at Westmead, Sydney, NSW;

[6]National Centre for Immunisation Research and Surveillance (NCIRS), Sydney, NSW;

[7]Barwon Health, University Hospital Geelong, Geelong, Victoria, Australia

[a]Corresponding author: soren.alexandersen@deakin.edu.au

Primer	Sequencing Target	Reference
**Calvert-6422-6446F**	GTNTAYARGATGATHATGATGGARA	23
**Calvert-7253-7275R**	YTTARTCAACACCATGGGAYYA	23
**Calvert-6512-6534F**	GAYTGGCACTTYATGATYAAYGC	23
**Calvert-7221-7243R**	ATNACMACWTCATAATCATCCAC	23
**Nix-Parecho-AN353**	GACAATAGTTTTGAAATNCANATHCCNTA	22
**Nix-Parecho-AN355**	CTCCAACTATAATGCCATARTGYTTRTARAANCC	22
**Nix-Parecho-AN357**	GAATAAAATGGTACTGANARNGTCATYTGYTC	22
**Nix-Parecho-AN369**	ACCAAGGTTGACAACATTTTYGGNMGNGC	22
**Parecho-SA-3541-3580F**	CACAGAAATTCTAGATAACGATTTGGTCAAATTCATAGTG	This study
**Parecho-SA-4290-4263R**	CCTTGTCCYGGTTTTTCCCGTWTCCAAA	This study
**Parecho-SA-7334-7294R**	TTTTTTTTTTTTGGTATGTCCAATATTCCAAATTAGTGTTC	This study
**Parecho-AC-3958-3986F**	TCAGTGGTTAGAGAGAAATAAGGAGCATG	This study
**Parecho-AC-4808-4780R**	CAAGTTGCCGTTTCCAGCAATYATAATTC	This study
**Parecho-AC-3979-4003F**	GGAGCATGTTTGTGCCATCTTGGAC	This study
**Parecho-AC-4669-4693R**	TTCCCAGCACTCTCCTGTGGCCAT	This study

Supplementary Table 1. *Sequencing primers used in this study.*

	V1 (V7121647)	V2 (V7133401)	V3 (V7140151)	V15 (V8117543)	V16 (V8140914)
**V1 (V7121647)**		79.1	77.67	76.96	77.67
**V2 (V7133401)**			88.36	89.31	87.89
**V3 (V7140151)**				89.79	99.05
**V15 (V8117543)**					89.31
**V16 (V8140914)**					

Supplementary Table 2. *Percentage similarity matrix of the 421nt alignment of the VP1 coding sequence of HPeV1 sequences.*

	W14 (CHW029)	W17 (CHW033)	V1 (V7121647)	V3 (V7140151)	V15 (V8117543)	V16 (V8140914)
**W14 (CHW029)**		90.08	79.32	86.5	85.65	86.29
**W17 (CHW033)**			77.64	85.65	85.86	85.44
**V1 (V7121647)**				78.69	78.9	78.9
**V3 (V7140151)**					86.92	98.52
**V15 (V8117543)**						85.44
**V16 (V8140914)**						

Supplementary Table 3. *Percentage similarity matrix of the 474nt of the VP1 coding sequence alignment of HPeV1 sequences*

HPeV Sequence Name	Abbreviated name	GenBank Accession	Reference
**Human parechovirus 3 strain Australia 2013-GL2015 isolate CSF19, complete genome**	CSF19	KY556671.1	20
**Human parechovirus 3 strain Australia 2013-GL2015 isolate FEC23, complete genome**	FEC23	KY556675.1	20
**Human parechovirus 3 strain Australia 2013-GL2015 isolate FEC22, complete genome**	FEC22	KY556674.1	20
**Human parechovirus 3 strain Australia 2013-GL2015 isolate FEC21, complete genome**	FEC21	KY556673.1	20
**Human parechovirus 3 strain Australia 2013-GL2015 isolate CSF01, complete genome**	CSF01	KY556659.1	20
**Human parechovirus 3 strain Australia 2013-GL2015 isolate FEC12, complete genome**	FEC12	KY556667.1	20
**Human parechovirus 3 strain Australia 2013-GL2015 isolate CSF08, complete genome**	CSF08	KY556665.1	20
**Human parechovirus 3 strain Australia 2013-GL2015 isolate NAS16, complete genome**	NAS16	KY556669.1	20
**Human parechovirus 3 strain Australia 2013-GL2015 isolate FEC20, complete genome**	FEC20	KY556672.1	20
**Human parechovirus 3 strain Australia 2013-GL2015 isolate CSF05, complete genome**	CSF05	KY556663.1	20
**Human parechovirus 3 strain Australia 2013-GL2015 isolate CSF04, complete genome**	CSF04	KY556662.1	20
**Human parechovirus 3 strain Australia 2013-GL2015 isolate FEC17, complete genome**	FEC17	KY556668.1	20
**Human parechovirus 3 strain Australia 2013-GL2015 isolate CSF06, complete genome**	CSF06	KY556661.1	20
**Human parechovirus 3 strain Australia 2013-GL2015 isolate NAS14, complete genome**	NAS14	KY556670.1	20
**Human parechovirus 3 strain Australia 2013-GL2015 isolate CSF07, complete genome**	CSF07	KY556664.1	20
**Human parechovirus 3 strain Australia 2013-GL2015 isolate CSF03, complete genome**	CSF03	KY556660.1	20
**Human parechovirus 3 strain Australia 2013-GL2015 isolate FEC10, complete genome**	FEC10	KY556666.1	20
**Human parechovirus 3 strain Yamagata 2011 lineage isolate NAS18 polyprotein gene, partial cds**	NAS18	KY556676.1	20
**Human parechovirus 3 isolate TW-03067-2011 polyprotein gene, complete cds**	KT626009	KT626009.1	27
**Human parechovirus 3 gene for polyprotein (VP0, VP3, VP1, 2A, 2B, 2C, 3A, 3B, 3C, 3D), partial cds, strain: 1873-Yamagata-2011**	AB759204	AB759204.1	21
**Human parechovirus 3 gene for polyprotein (VP0, VP3, VP1, 2A, 2B, 2C, 3A, 3B, 3C, 3D), partial cds, strain: 1585-Yamagata-2011**	AB759207	AB759207.1	21
**Human parechovirus 3 gene for polyprotein (VP0, VP3, VP1, 2A, 2B, 2C, 3A, 3B, 3C, 3D), partial cds, strain: 1924-Yamagata-2011**	AB759205	AB759205.1	21

Supplementary Table 4. *HPeV sequences from GenBank used in the recombination, selection and phylogenetic analyses in this study.*

Sample Set	Genome region	# Sequences	Nucleotide Length	Codons	GARD	SLAC p0.05	FEL p0.01	FEL 0.05	REL 100	Non-synonymous/ Synonymous	Transition/ Transversion	Sites with Nucleotide Differences	% NT diffs	Codons with Amino acid differences	% AA Differences
**Australian Recombinant HPeV3 2013-2018**	Polyprotein	40	6531	2177	Nil	Pos 0 codons, Neg 40 codons	Pos 0 codons, Neg 54 codons	Pos 4 codons, Neg 63 codons	8 Pos sites (all in NS proteins)	0.1163	9.45	332	5.083	51	2.343
**Australian Recombinant HPeV3 2013-2018**	Capsid	40	2313	771	Nil	Pos 0 codons, Neg 1 codon	Pos 0 codons, Neg 1 codon	Pos 0 codons, Neg 5 codons	0 Pos Sites	0.0283	9.77	89	3.848	6	0.778
**Australian Recombinant HPeV3 2013-2018**	Non-structural Proteins	40	4218	1406	Nil	Pos 0 codons, Neg 3 codons	Pos 0 codons, Neg 1 codon	Pos 0, Neg 7 codons	8 Pos sites	0.114	8.578	243	5.761	45	3.201

Supplementary Table 5. *Results of recombination and selection analysis from the Datamonkey webserver for the coding and noncoding regions of the Australian recombinant HPeV type 3 sequences.*

Case	Amino Acid residue 1359	Amino acid residue 1366	# basic amino acids variable sites
Case W1	K	R	2
Case W2	N	R	1
Case W3	K	Q	1
Case W4	K	R	2
Case W5	K	Q	1
Case W6	N	R	1
Case W7	K	R	2
Case W8	K	Q	1
Case W9	K	Q	1
Case W10	K	R	2
Case W11	K	R	2
Case W12	K	R	2
Case W13	K	R	2
Case W14	HPeV1		
Case W15	N	R	1
Case W16	N	R	1
Case W17	HPeV1		
Case V1	HPeV1		
Case V2	HPeV1		
Case V3	HPeV1		
Case V4	K	R	2
Case V5	N	R	1
Case V6	N	R	1
Case V7	K	R	2
Case V8	N	R	1
Case V9	N	R	1
Case V10	N	R	1
Case V11	HPeV3 Taiwan lineage		
Case V12	HPeV3 Taiwan lineage		
Case V13	N	R	1
Case V14	K	R	2
Case V15	HPeV1		
Case V16	HPeV1		

Supplementary Table 6. *The amino acid residues at codons 1359 and 1366 within the polyprotein of Australian recombinant HPeV3 viruses. These changes occur within the 2C protein of HPeV3. The viruses had 1 or 2 basic amino acid residues at these positions.*

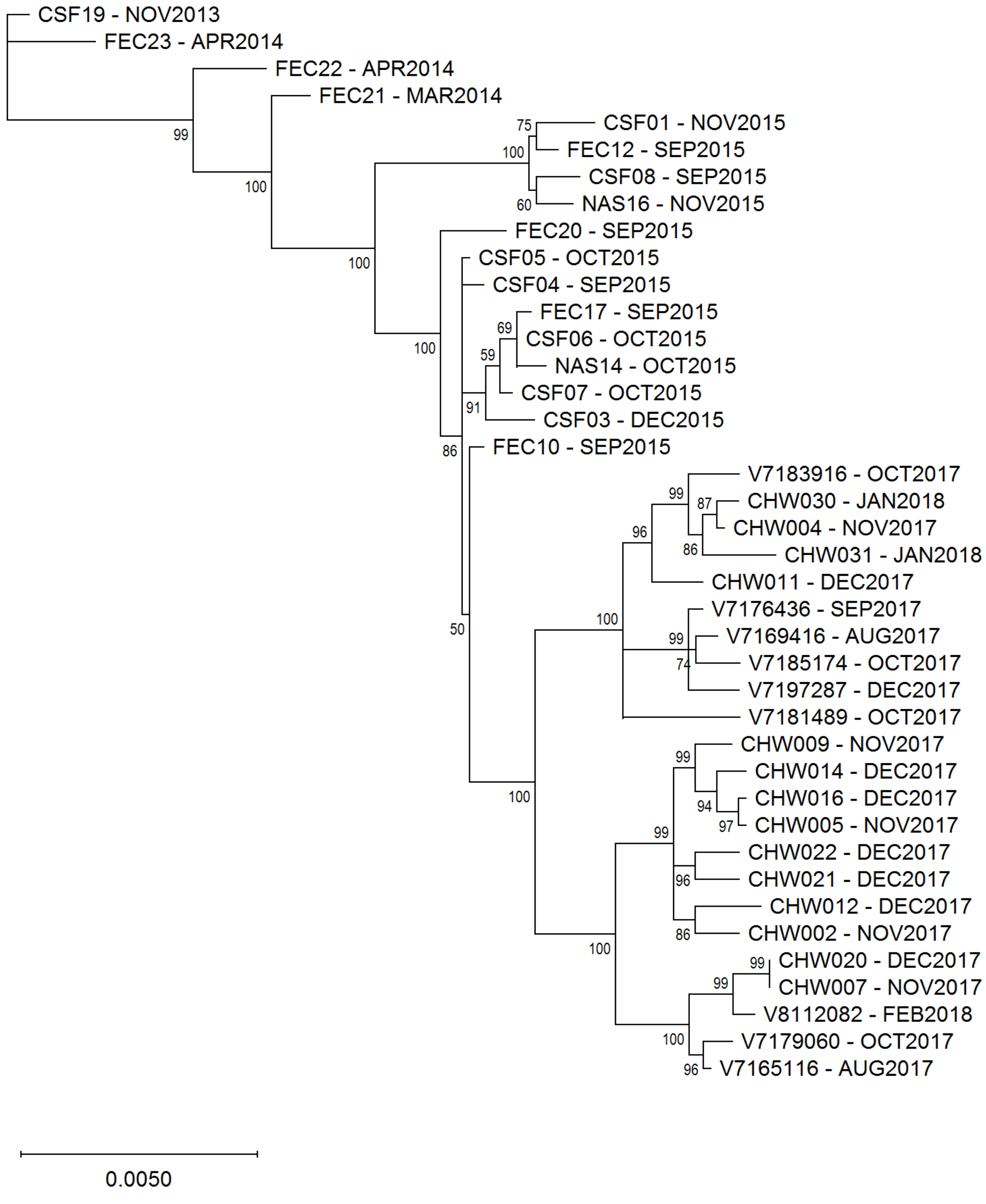

Supplementary Figure 1. *The phylogenetic tree of the complete nucleotide sequence (6534nt) of the polyprotein gene of 23 of the HPeV type 3 viruses detected in the Australian 2017 epidemic and similar viruses identified in the previous Australian epidemics of 2013 and 2015. Sequences were aligned by MUSCLE and phylogenetic analysis was performed in MEGA X using the Tamura 3-parameter model and the maximum likelihood method. Bootstrap testing involved 1000 replicates to determine the reliability of the inferred tree and branch length is scaled proportional to the number of nucleotide substitutions. The sample name and Month and year of sample collection are shown.*

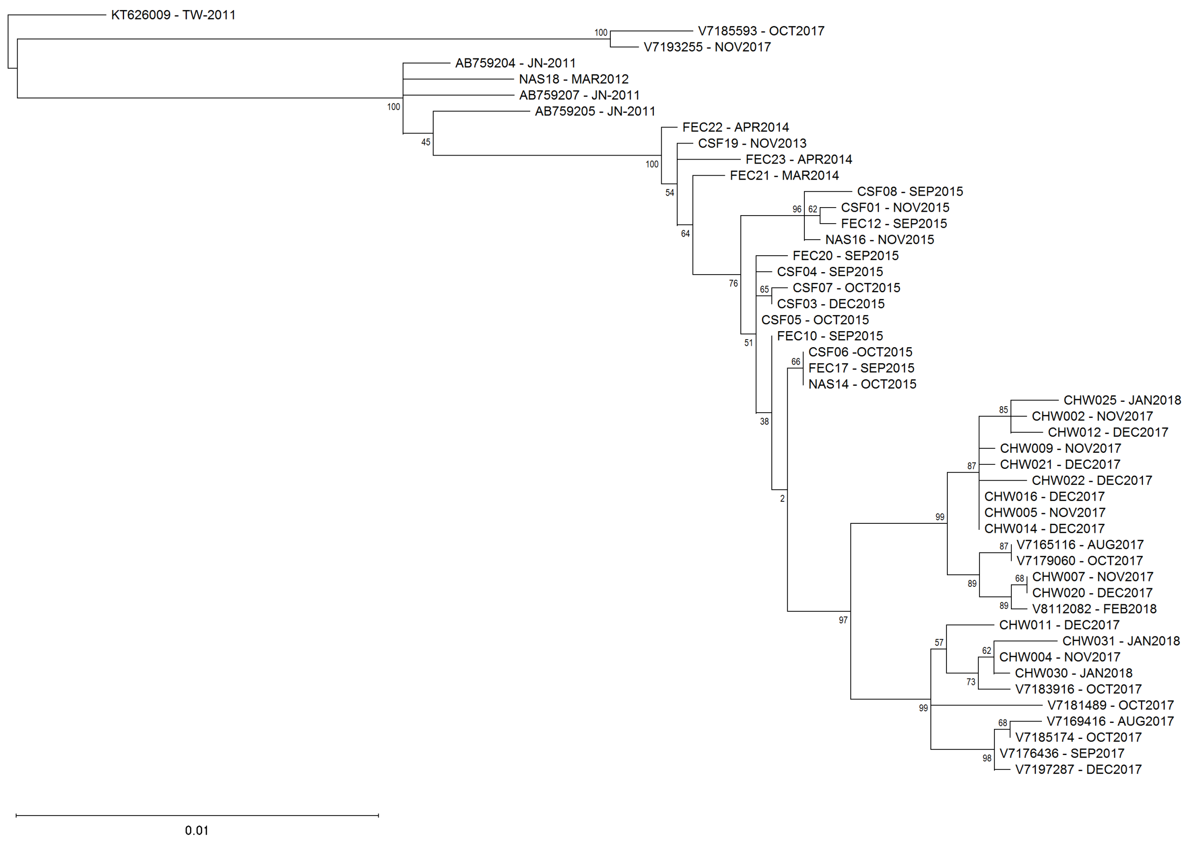

Supplementary Figure 2. *The phylogenetic tree of the complete capsid nucleotide sequence (2313nt) of the HPeV type 3 viruses identified in Australia 2012-2018 and the related representative viruses identified overseas. Sequences were aligned by MUSCLE and the phylogenetic analysis was performed in MEGA X using the Tamura 3-parameter model and the maximum likelihood method. Bootstrap testing involved 1000 replicates to determine the reliability of the inferred tree and branch length is scaled proportional to the number of nucleotide substitutions. The sample name and month and year of sample collection are shown. The structural protein genes of all Australian HPeV type 3's except V7185593 and V7193255 belonged to a Yamagata-2011 HPeV type lineage.*

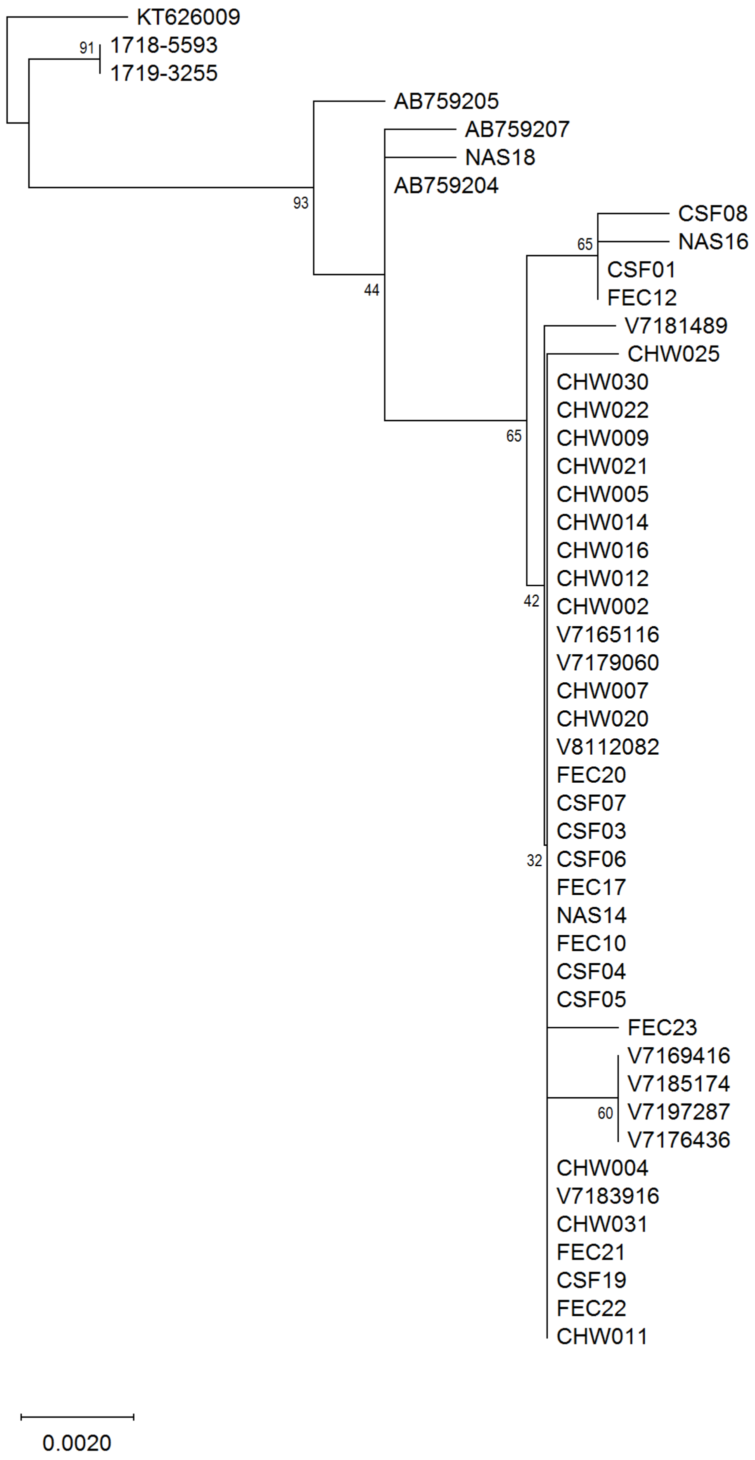

Supplementary Figure 3. *The phylogenetic tree of the complete capsid amino acid sequence (771AA) of the HPeV type 3 viruses identified in Australia 2012-2018 and the related representative viruses identified overseas. Sequences were aligned by MUSCLE and phylogenetic analysis was performed in MEGA X using the Jones-Taylor-Thornton model and the maximum likelihood method. Bootstrap testing involved 500 replicates to determine the reliability of the inferred tree and branch length is scaled proportional to the number of amino acid substitutions. The tree was rooted on the oldest sequence (KT626009). The capsid protein showed very little variation in Australian recombinant HPeV type 3 viruses from the 2015 and the 2017 epidemics.*

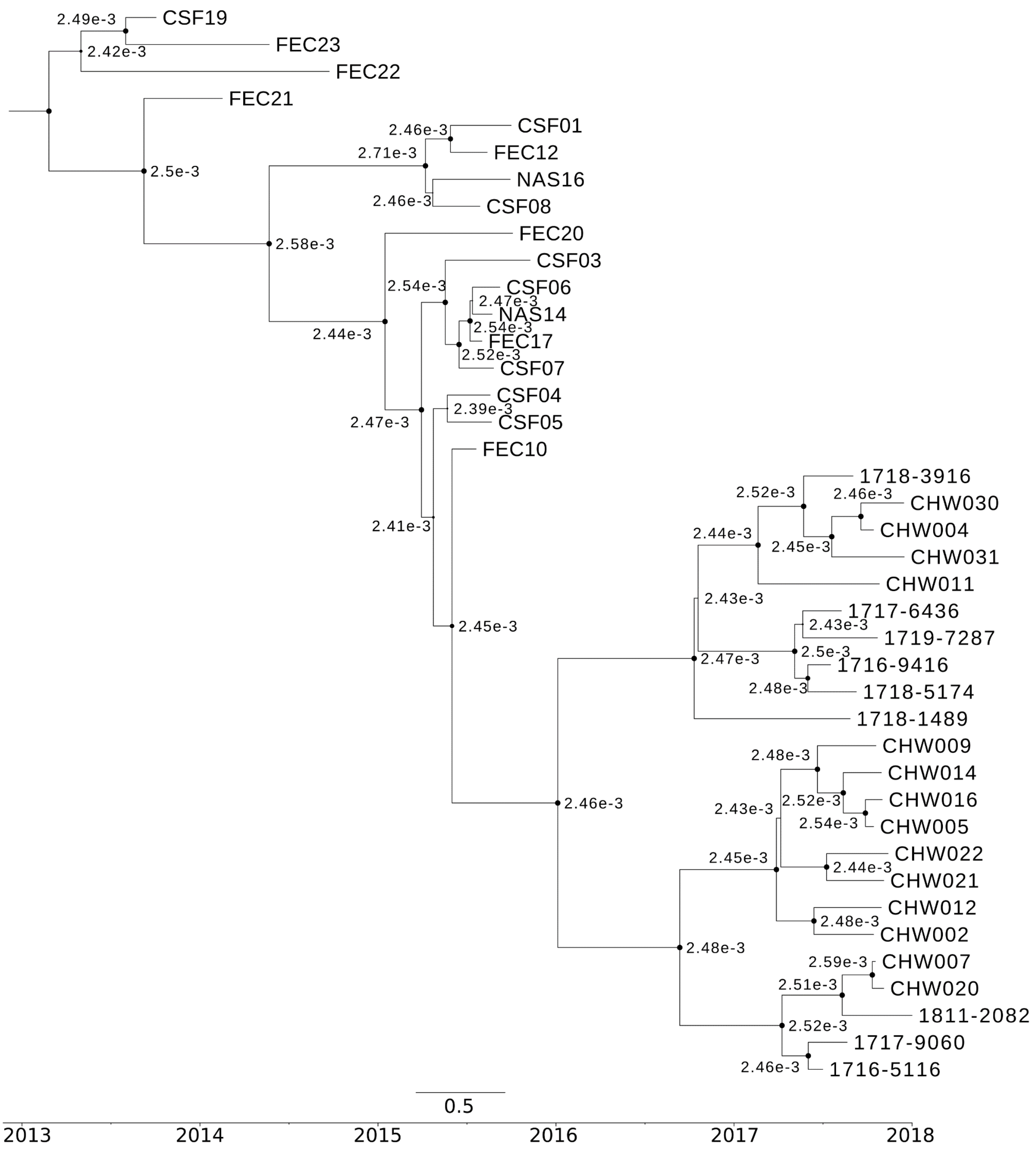

Supplementary Figure 4. *The maximum clade credibility tree of the complete nucleotide sequence (6534nt) of the polyprotein gene of 23 of the HPeV type 3 viruses detected in the Australian 2017 epidemic and similar viruses identified in the previous Australian epidemics of 2013 and 2015. Sequences were aligned by MUSCLE and phylogenetic analysis was performed in Beast 2.5.2 using the HKY model with a gamma distribution, a Markov Chain Monte Carlo chain length of 50 million and a relaxed evolutionary clock. Node circles are scaled proportional to the posterior probability and node rates are shown.*

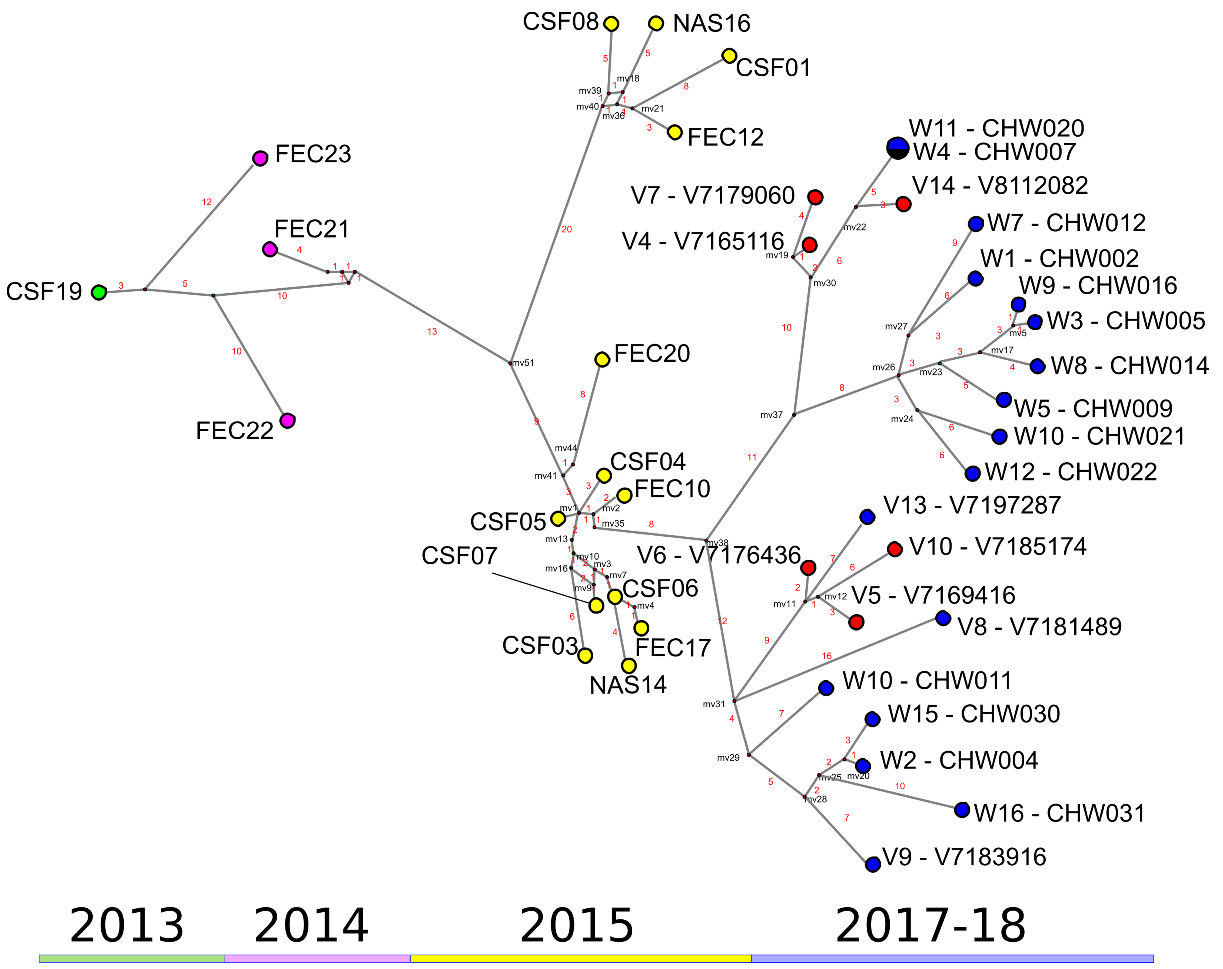

Supplementary Figure 5. *The minimum spanning network of the polyprotein nucleotide sequences of the recombinant HPeV type 3 sequences from the 2013, 2015 and 2017 epidemics (year of sampling is shown in scale along the bottom of the network). Within the 2017 epidemic there were three networks of cases identified among the HPeV type 3 viruses sequenced. Red cases were from Victoria 2017-18; blue cases were from NSW/ACT 2017-18; yellow cases from the 2015 epidemic and green and pink from the 2013-14 epidemic.*

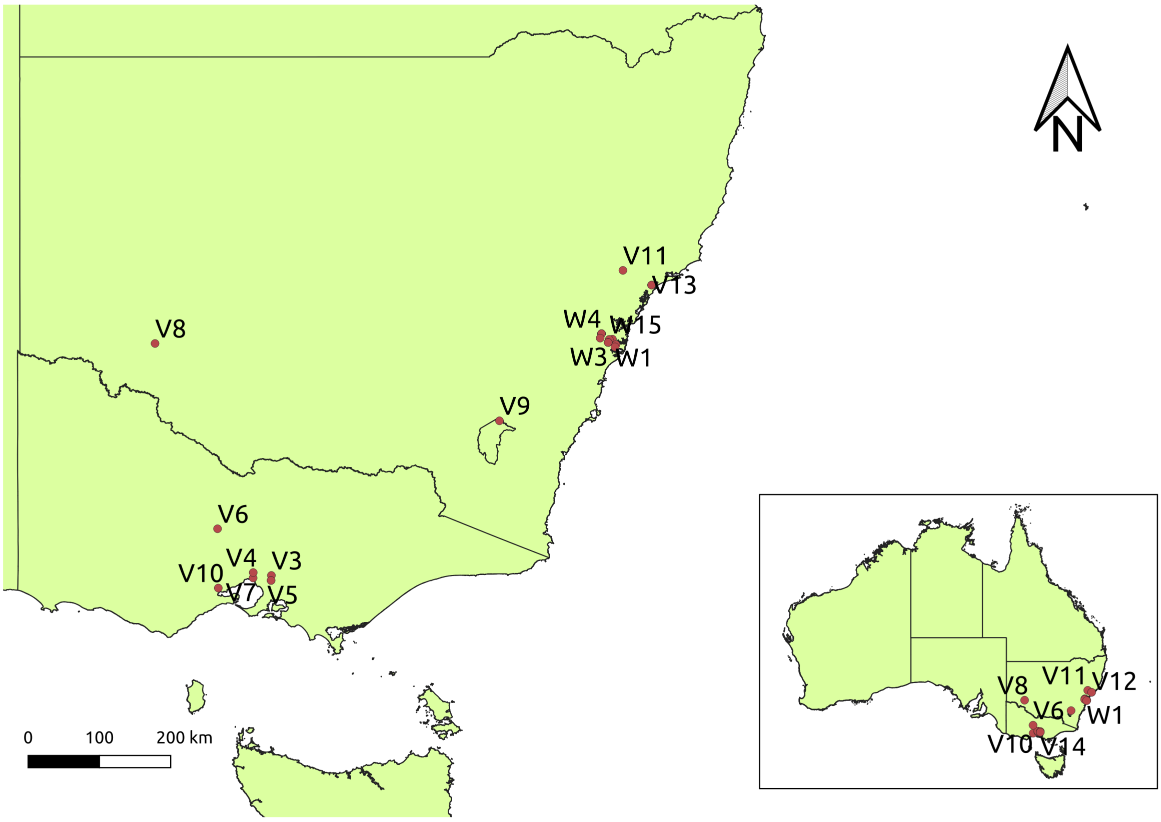

Supplementary Figure 6. *The 33 HPeV cases from the 2017 epidemic in south eastern Australia plotted to the closest statisitical division. Several areas within metropolitan regions had multiple cases and some labels have been removed for clarity. Inset shows case locations on map of Australia.*

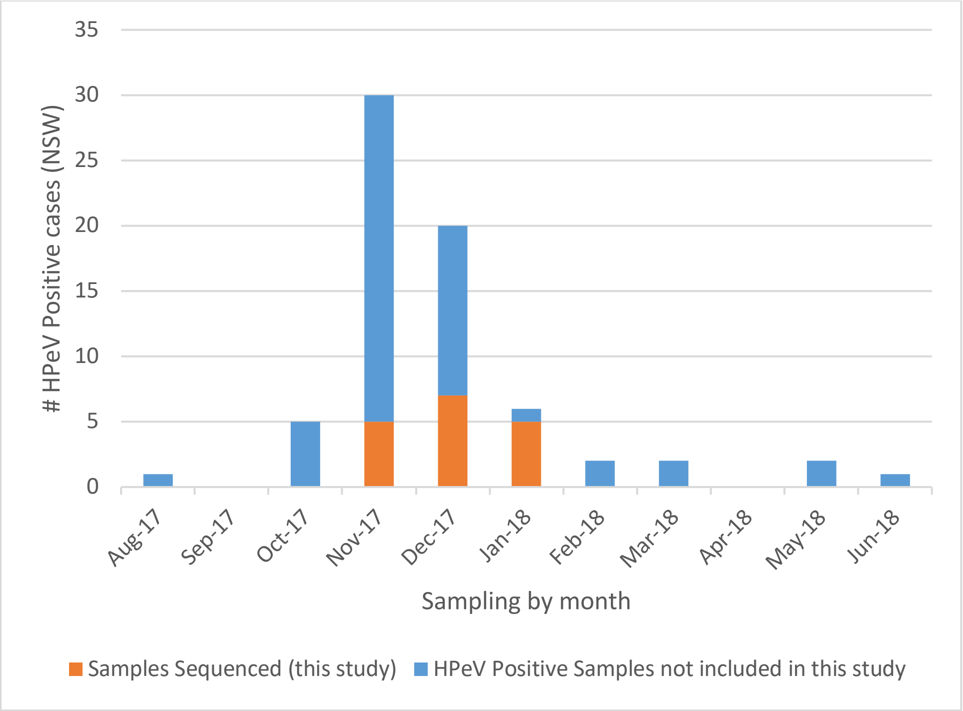

Supplementary Figure 7. *The time of collection of the 17 human parechovirus cases from New South Wales (NSW) sentinel site included in this study, as a proportion of the total (69) cases from that site identified between July 2017 and Jun 2018. The samples in this study represent 25% of the total cases, and were collected during and after the peak of the epidemic in this state.*
